# Phase-contrast computed tomography for quantification of structural changes in lungs of asthma mouse models of different severity

**DOI:** 10.1107/S1600577515006177

**Published:** 2015-06-17

**Authors:** Christian Dullin, Emanuel Larsson, Giuliana Tromba, Andrea M. Markus, Frauke Alves

**Affiliations:** a Institute of Diagnostic and Interventional Radiology, University Medical Center Goettingen, Robert Koch Strasse 40, Goettingen, Lower Saxony 37075, Germany; b Elettra-Sincrotrone Trieste, Strada Statale 14, km 163,5 in AREA Science Park, Basovizza (Trieste) 34149, Italy; c Department of Architecture and Engineering, University of Trieste, Trieste, Italy; d Department of Physics, Chemistry and Biology, Linkoeping University, SE-581 83 Linkoeping, Sweden; e Department of Haematology and Medical Oncology, University Medical Center Goettingen, Robert Koch Strasse 40, Goettingen, Lower Saxony 37075, Germany; f Department of Molecular Biology of Neuronal Signals, Max Planck Institut for Experimental Medicine, Hermann-Rein-Strasse 3, Goettingen, Lower Saxony 37075, Germany

**Keywords:** phase-contrast CT, single-distance phase retrieval, lung imaging, asthma mouse models

## Abstract

Lung imaging in mouse disease models is crucial for the assessment of the severity of airway disease but remains challenging due to the small size and the high porosity of the organ. Synchrotron inline free-propagation phase-contrast computed tomography (CT) with its intrinsic high soft-tissue contrast provides the necessary sensitivity and spatial resolution to analyse the mouse lung structure in great detail. Here, this technique has been applied in combination with single-distance phase retrieval to quantify alterations of the lung structure in experimental asthma mouse models of different severity. In order to mimic an *in vivo* situation as close as possible, the lungs were inflated with air at a constant physiological pressure. Entire mice were embedded in agarose gel and imaged using inline free-propagation phase-contrast CT at the SYRMEP beamline (Synchrotron Light Source, ‘Elettra’, Trieste, Italy). The quantification of the obtained phase-contrast CT data sets revealed an increasing lung soft-tissue content in mice correlating with the degree of the severity of experimental allergic airways disease. In this way, it was possible to successfully discriminate between healthy controls and mice with either mild or severe allergic airway disease. It is believed that this approach may have the potential to evaluate the efficacy of novel therapeutic strategies that target airway remodelling processes in asthma.

## Abstract 2

Synchrotron inline phase-contrast computed tomography in combination with single-distance phase retrieval enables quantification of morphological alterations in lungs of mice with mild and severe experimental allergic airways disease in comparison with healthy controls.

## 1. Introduction   

Mouse lung disease models are widely used in preclinical asthma research (Bates *et al.*, 2009 ▸). Despite certain limitations (Epstein, 2004 ▸), they are the method of choice to gain insight into the pathomechanism of this complex multifactorial disorder and to evaluate novel therapeutic concepts (Markus *et al.*, 2014 ▸). However, the smallness of the lung, its high porosity and the minor alterations in the lung structure caused by asthma render classical imaging strategies like medical ultrasound, MRI and computed tomography (CT) extremely challenging. Synchrotron-radiation-based CT has been proven very effective in lung imaging (Lewis, 1997 ▸) and can provide spatial resolutions down to the sub-micrometre level. Studies conducted at the European Synchrotron Facility by Bayat *et al.* (2008 ▸) showed that the *K*-edge absorption technique using xenon as contrast agent is very effective for *in vivo* imaging of lung functionality. However, the used pixel size of 350 µm × 350 µm did not allow an accurate evaluation of alterations in pulmonary morphology. Moreover, phase-sensitive techniques such as free-propagation inline phase-contrast CT and grating interferometry imaging with their improved soft-tissue contrast (Beltran *et al.*, 2011 ▸) have already been successfully applied for lung imaging by various groups such as Yagi, Kitchen, Hooper and Lewis (Kitchen *et al.*, 2004 ▸; Hooper *et al.*, 2007 ▸; Lewis *et al.*, 2005 ▸; Yagi *et al.*, 1999 ▸) to name but a few. Here, we used synchrotron radiation inline phase-contrast CT (XPCT) in combination with single-distance phase-retrieval algorithms for anatomical imaging of mouse lungs, as it provides a detailed high-contrast depiction of biological soft tissue while requiring only one tomographic acquisition. This approach has already been proven beneficial in lung imaging by analysing the phase shift of the X-ray incident beam within the sample (Parsons *et al.*, 2008 ▸; Kitchen *et al.*, 2005 ▸; Yong *et al.*, 2009 ▸). Moreover, in a pilot study we demonstrated that with XPCT the contrast-to-noise ratio (CNR) in lung imaging can be increased by at least a factor of ten when compared with classical absorption-based CT (Mohammadi *et al.*, 2014 ▸). In order to test the capability of XPCT to distinguish between minor and major lung alterations we chose two experimental allergic airway disease mouse models of different severity, one that resembles mild acute allergic asthma (Dullin *et al.*, 2015 ▸) expressing only minor morphological changes and one that mimics severe asthma with dominant alterations of the lung structure (Nabe *et al.*, 2005 ▸). Both models were previously reported by us and others (Markus *et al.*, 2014 ▸; Dullin *et al.*, 2015 ▸; Biffi *et al.*, 2013 ▸; Bosnjak *et al.*, 2014 ▸) and exhibit reproducible numbers of eosinophils in bronchoalveolar lavage as well as a consistent amount of cell infiltration in histology.

We show that XPCT can discriminate between the two airway disease models and that this technique provides the necessary sensitivity for quantitative analysis of structural differences in the lungs by comparing parameters like soft-tissue content and changes in the lung tissue composition, parameters that correlate with typical hallmarks of asthma and thereby with the severity of the disease.

## 2. Methods   

Female BALB/c mice (4–6 weeks old) were purchased from Harlan Laboratories and maintained with *ad libitum* food and water. Two experimental allergic airway disease models of different severity were generated to mimic ‘mild’ acute allergic asthma (MAA) (Markus *et al.*, 2014 ▸; Biffi *et al.*, 2013 ▸; Dullin *et al.*, 2015 ▸) and ‘severe’ acute allergic asthma (SAA) (Nabe *et al.*, 2005 ▸). For induction of MAA, mice were sensitized twice intraperitoneally (i.p.) with 10 µg ovalbumin (OVA) in 200 µl phosphate-buffered saline (PBS) on days 0 and 21. For SAA, mice were sensitized on days 0 and 14 i.p. with a mixture of 50 µg OVA and 0.5% of aluminium hydroxide adjuvant (Invivogen, San Diego, USA) in a volume of 200 µl PBS, as well as intranasally (i.n.) with 50 µg OVA in 25 µl PBS. In order to provoke an acute allergic reaction, mice were treated i.n. either with a solution of 100 µg OVA/50 µl PBS/mouse (MAA, at days 28 and 29) or with a solution of 250 µg OVA/50 µl PBS/mouse (SAA, at days 28, 29, 30, and 33). The control group (CN) was composed of mice which received PBS only, following the schedule of the MAA model. Each group contained four mice. All animal *in vivo* procedures were performed at the University Medical Center Goettingen, Germany, in compliance with the guidelines of the European (86/609/EEC) and the German ethical laws and were approved by the administration of Lower Saxony, Germany.

Mice were sacrificed two days after the last challenging step, *i.e.* on day 31 (MAA, CN) and on day 35 (SAA). In order to mimic the *in vivo* properties of the lung as close as possible, samples were prepared for the phase-contrast CT analysis as described before (Dullin *et al.*, 2015 ▸) by performing a tracheotomy on the sacrificed mice, followed by inflation of the lungs with air at a constant pressure of 30 cm water column. Finally, tracheas were tied up and the whole mice were embedded in 1% agarose gel in 30 ml falcon tubes.

The samples were imaged at the SYRMEP beamline (Synchrotron Light Source ‘Elettra’, Trieste, Italy) with the following parameters: X-ray energy *E* = 22 keV, 1800 projections in a 360° acquisition mode using an exposure time per projection of 2.4 s. For detection, we utilized a water-cooled CCD camera (Photonic Science, model VHR) with a 4008 × 2672 full frame in 2 × 2 binning mode (resulting in a pixel size of 9 µm × 9 µm), coupled to a gadolinium oxysulfide scintillator placed on a fibre optic taper. The agarose embedded mice were mounted in an upright position and their central lung part (∼4 mm in height) was imaged. A sample-to-detector distance of 30 cm was chosen to allow for inline phase-contrast measurements. In order to decouple the phase shift from the absorption effect, a single-distance phase-retrieval algorithm [TIE_Hom (Gureyev *et al.*, 2009 ▸; Paganin *et al.*, 2002 ▸), *X-tract* software package, CIRS, Australia] was applied to the projection images (Mohammadi *et al.*, 2014 ▸) before reconstruction using a classical filtered back-projection algorithm (FBP). This resulted in three-dimensional (3D) data sets, predominately representing the real part of the complex refractive index, demonstrating a high CNR as well as a good edge quality (Mohammadi *et al.*, 2014 ▸).

CT imaging results were correlated to histology. For this purpose, lung samples were obtained from a further set of OVA-induced asthmatic and control mice following the same asthma induction protocols. Excised lungs were fixed in 10% buffered formalin and embedded in paraffin. 3 µm-thick paraffin lung sections containing main stem bronchi were obtained and hematoxylin-eosin (H&E) stained for 2 min. Finally, these stained sections were dehydrated using an ascending alcohol series and xylol followed by mounting with DePex (Serva, Heidelberg, Germany). An Axioskop 2 (Carl Zeiss Microscopy GmbH, Jena, Germany) microscope in combination with a Leica DC 100 camera (Leica, Switzerland) was used for visualization of the stained sections.

For quantification of differences in the water content of the lung tissue from asthmatic and healthy mice an additional cohort of age-matched mice was used (MAA, *N* = 6; SAA, *N* = 5; CN, *N* = 6), that were immunized and challenged following the same protocol as the mice analysed by CT and histology. Lungs from these mice were excised at the same time point on day 31 (MAA, CN) and on day 35 (SAA). One half of the lung was fixed in 10% buffered formalin for later histological verification of the presence or absence of asthma. The other half was weighed directly after explantation (wet) and after a 24 h drying process (dry) using a vacuum concentrator (SpeedVac^TM^, ThermoSientific).

## 3. Results and discussion   

**Figure 1:**
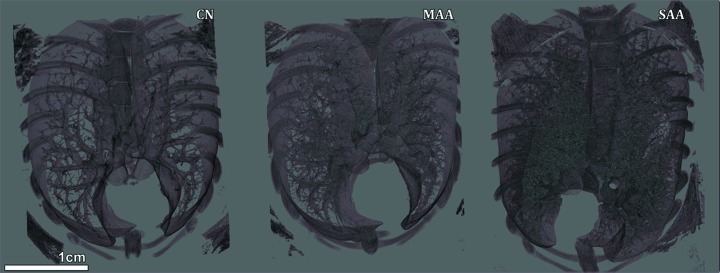
Volume rendering representations of the obtained XPCT results for lungs from a healthy control mouse (CN), a mouse from the mild (MAA) and a mouse from the severe (SAA) experimental allergic airway model. Increased soft-tissue content is clearly visible within lungs in correlation with increasing severity of asthma.

**Figure 2:**
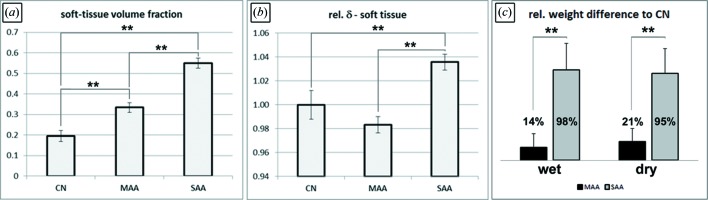
(*a*) The measured soft-tissue volume fractions obtained in eight VOIs per mouse are shown. Note that the same trend of increasing soft-tissue content seen in Fig. 1 ▸ can be found here. (*b*) The relative mean δ-values of the lung soft tissue (normalized to the control group CN) are demonstrated. An increase of the relative δ-value is found in lungs of mice with severe asthma (SAA). In contrast, lungs of mice with mild asthma (MAA) demonstrate slightly reduced relative δ-values. (*c*) Relative difference of the lung weight of MAA (*N* = 6) and SAA (*N* = 5) compared with CN (*N* = 6) (additional cohort of mice) directly after explantation (wet) and after being dried for 24 h (dry). Lungs of SAA mice weighed almost twice as much as lungs of CN with a small reduction in dry weight, indicating that the difference is equally related to more cells and higher water content. The relative weight difference of MAA increases from 14% to 21% from the wet to dry state, indicating that this effect is much smaller than in SAA and is slightly more related to an increase in the number of cells within the lung than to a higher water content. The error bars represent the standard deviation of the respective values within the different groups. ** indicates a *p*-value of a one-way ANOVA test of less than 0.01 and therefore a significant difference.

By applying inline phase-contrast CT in combination with single-distance phase retrieval we found that the differences in severity of the disease in these two experimental allergic airway models in comparison with healthy controls are reflected in an increase in the soft-tissue content of the lung. Fig. 1 ▸ shows representative images of the soft-tissue–air interface of lungs from one mouse of each group (CN, MAA and SAA) rendered in 3D using the same parameters. The observed increase in the soft-tissue content correlates with the severity of the analysed models. In order to quantify these alterations in the lung structure, eight non-overlapping volumes-of-interests (VOIs) of 2 mm × 2 mm × 2 mm were placed uniformly in the peripheral region of the lung in order to depict comparable alveolar structures and ensure reproducibility of the measurement in different samples. The grey value histogram of such a VOI shows two dominant peaks representing air and soft tissue. The mean value between these two peaks was chosen as a threshold to discriminate air from soft tissue and the threshold was kept constant for all VOIs and samples. The volume ratio (Vol.Ratio) of the soft-tissue component was calculated by dividing the total volume of all voxels within the soft-tissue threshold range within the VOI by the VOI volume. The values of the eight VOIs per sample were averaged and the results are shown in Fig. 2(*a*) ▸. The three groups (CN, MAA and SAA) are clearly distinguishable by a statistically significant (one-way ANOVA test, *p*-value < 0.01; Fig. 2*a* ▸) increase in the soft-tissue Vol.Ratio in correlation with the severity of the analysed models (0.2 in CN, 0.33 in MAA and 0.55 in SAA).

The success of our imaging approach to discriminate between diseased and healthy mice is vastly dependent on our preparation scheme (Dullin *et al.*, 2015 ▸) which is designed to minimize alterations in the samples during the scanning procedure. Inflating the lung with air at a constant pressure results in an increased total lung volume (soft-tissue and air, our own previous observation) and is most likely caused by reduced elasticity of the lung tissue in asthmatic mice, already described by Gelb *et al.* (2002 ▸). Therefore, the increased soft-tissue volume ratio, which was found to be significantly higher in the diseased mice, still underestimates the effect *in vivo*. These *ex vivo* findings must not be confused with the so-called ‘air-trapping’ effect, a symptom often seen in asthmatic patients and used as a clinical parameter in CT-based asthma diagnosis. ‘Air trapping’ actually increases the air content in certain lung areas at the expiration phase due to a reduced ability to expel air (Busacker *et al.*, 2009 ▸).

Another important aspect that has to be considered is that asthma is accompanied by an increased mucus production. The capabilities of our imaging strategy are limited in terms of discriminating mucus from soft tissue. Therefore, the analysed total amount in the tissue content of the asthmatic lungs may be somewhat smaller if mucus is present. However, the measurement of the air content of the asthmatic lungs is unaffected by this limitation and suggests that the analysed Vol.Ratio of non-air components within the lung represents a good parameter for the characterization of the severity of the allergic reaction in each lung sample.

**Figure 3:**
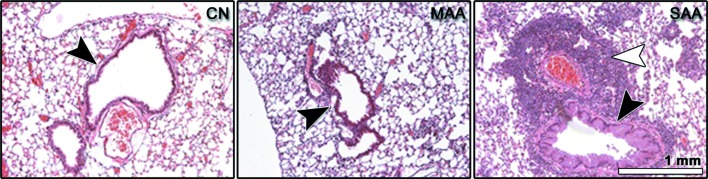
H&E stained histological sections of a control lung (CN), of a lung from a mouse taken from the mild (MAA) and from the severe (SAA) experimental allergic lung disease model, all sacrificed four days after the last OVA challenge. Lung sectiοns of mice from MAA and SAA show airway wall thickening (bronchial wall indicated by black arrow heads) and lungs of mice with SAA contain areas of high cell density (white arrow head) which are absent in CN and MAA, all parameters known to be typical hallmarks of asthma.

In order to validate the observed increase in soft-tissue content in asthmatic lungs, sections of lungs explanted four days after the last challenge were histologically analyzed. Fig. 3 ▸ shows representative results for one sample of each group. In accordance with other reports, we found an increased thickness of the bronchial walls, which is associated with the severity of the allergic inflammatory reaction in mice in these asthmatic mouse models (Dullin *et al.*, 2015 ▸). In addition, areas with a high cell density as shown in Fig. 3 ▸ can only be found in SAA samples, confirming the strongly increased soft-tissue Vol.Ratio depicted by phase-contrast CT in SAA.

The application of a phase-retrieval algorithm results in 3D data sets which predominately express the real part (δ-value) of the refractive index within the samples (Gureyev *et al.*, 2013 ▸). Since the δ-value is characteristic of a certain element, the mean grey value of the segmented soft tissue contains information about the chemical tissue composition. Note that, whereas the imaginary part of the refractive index can quite reliably be estimated by the used phase-retrieval algorithm (TIE-Hom), the δ-value depends on the chosen δ-to-β ratio as indicated by Gureyev *et al.* (2013 ▸). Therefore, there is no direct relation of the calculated grey values to the δ-value. However, since the same δ-to-β ratio was used for all samples, a similar grey level can be expected in all groups if the lung soft tissue has the same composition. Here, in order to facilitate the comparison between the three groups, the individual average grey values for the eight VOIs analysed per sample were normalized to the mean value of the CN group as shown in Fig. 2(*b*) ▸.

A slightly (2%) reduced mean δ-value of lung soft tissue was found in MAA and an (4%) increased mean δ-value in SAA mice [one-way ANOVA test, *p*-value < 0.01, Fig. 2(*b*) ▸], pointing to differences in the airway remodelling mechanism in these two experimental allergic airway disease models. In order to further analyse these findings, lungs of an additional cohort of mice were explanted and the wet and dry weight of each left lung lobe was measured for each group (CN, MAA, SAA). In Fig. 2(*c*) ▸ the results of this experiment are displayed as the relative difference to the average left lung lobe weight of CN (wet and dry, respectively). The wet weight reflects both the weight of the water and the cells in the lung, whereas the dry weight reflects the cells only. MAA shows a 14% and SAA a 98% larger wet weight than CN, which changes to 21% and 95%, respectively, after the drying process. This indicates that the 14% higher wet weight of the lungs from MAA is rather related to the presence of more cells than to a higher water content, since the difference increased when the lungs were dried. In contrast, in SAA the increased weight of 98% must be related to both the presence of more cells and increased water content, as the relative weight difference to controls changes only marginally. These results indeed suggest that the two allergic airway disease models lead to different tissue compositions of the lung.

To compare these results with the obtained phase-contrast CT δ-values (Fig. 2*b* ▸), the soft-tissue volume fraction also has to be considered. MAA shows an about 65% and SAA an about 175% increased soft-tissue volume fraction compared with CN. Therefore, the ratio between the increased amount of cells and increased soft-tissue volume fraction is 0.32 in MAA (21%/65%) and 0.54 in SAA (95%/175%) indicating that the cells in MAA are more loosely packed than in SAA. This effect may cause the different trends in the observed average δ-values in MAA and SAA. The packaging of cells may be related to the extracellular matrix (ECM) and it is known that in asthma an increased percentage of areas of collagen can be found in the ECM (Weitoft *et al.*, 2014 ▸), which most likely causes the swelling and reduction in lung tissue elasticity. Therefore, our results indicate a difference in the ECM components between mild and severe asthma, a theory which needs to be studied in more detail.

In summary, we show that synchrotron inline phase-contrast CT with a pixel size of about 9 µm × 9 µm in combination with a single-distance phase-retrieval algorithm provides the necessary image quality for discrimination and quantification of variable alterations in the lung structure. Our results show that, based on quantification of structural alterations within the lung, even mice from a weak allergic airway disease model can be significantly discriminated from controls and stronger allergic airway disease and highlights the robustness and versatility of the presented approach. Inline phase-contrast CT in combination with single-distance phase retrieval therefore represents a valuable tool for the characterization of morphological changes in allergic airway disease mouse models and may aid the analysis of the efficacy of novel therapeutic approaches.

## References

[bb1] Bates, J. H., Rincon, M. & Irvin, C. G. (2009). *Mol. Physiol.* **297**, L401–L410. 10.1152/ajplung.00027.2009 PMC2739768 19561139

[bb2] Bayat, S., Porra, L., Suhonen, H., Janosi, T., Strengell, S., Habre, W., Petak, F., Hantos, Z., Suortti, P. & Sovijärvi, A. (2008). *Eur. J. Radiol.* **68**, S78–S83. 10.1016/j.ejrad.2008.04.043 18606518

[bb3] Beltran, M. A., Paganin, D. M., Siu, K. K. W., Fouras, A., Hooper, S. B., Reser, D. H. & Kitchen, M. J. (2011). *Phys. Med. Biol.* **56**, 7353–7369. 10.1088/0031-9155/56/23/002 22048612

[bb5] Biffi, S., Dal Monego, S., Dullin, C., Garrovo, C., Bosnjak, B., Licha, K., Welker, P., Epstein, M. M. & Alves, F. (2013). *PLoS One*, **8**, e57150. 10.1371/journal.pone.0057150 PMC3578827 23437332

[bb6] Bosnjak, B., Tilp, C., Tomsic, C., Dekan, G., Pieper, M. P., Erb, K. J. & Epstein, M. M. (2014). *Pulm. Pharmacol. Ther.* **27**, 44–51. 10.1016/j.pupt.2013.09.004 24090641

[bb7] Busacker, A., Newell, J. D. Jr, Keefe, T., Hoffman, E. A., Cook Granroth, J., Castro, M., Fain, S. & Wenzel, S. (2009). *CHEST J.* **135**, 48. 10.1378/chest.08-0049 PMC2849984 18689585

[bb8] Dullin, C., dal Monego, S., Larsson, E., Mohammadi, S., Krenkel, M., Garrovo, C., Biffi, S., Lorenzon, A., Markus, A., Napp, J., Salditt, T., Accardo, A., Alves, F. & Tromba, G. (2015). *J. Synchrotron Rad.* **22**, 143–155. 10.1107/S1600577514021730 PMC4294027 25537601

[bb9] Epstein, M. M. (2004). *Int Arch Allergy Immunol.* **133**, 84–100. 10.1159/000076131 14726635

[bb10] Gelb, A. F., Licuanan, J., Shinar, C. M. & Zamel, N. (2002). *Chest*, **121**, 715–721. 10.1378/chest.121.3.715 11888951

[bb11] Gureyev, T. E., Mayo, S. C., Myers, D. E., Nesterets, Y., Paganin, D. M., Pogany, A., Stevenson, A. W. & Wilkins, S. W. (2009). *J. Appl. Phys.* **105**, 102005.

[bb12] Gureyev, T., Mohammadi, S., Nesterets, Y., Dullin, C. & Tromba, G. (2013). *J. Appl. Phys.* **114**, 144906.

[bb13] Hooper, S. B., Kitchen, M. J., Wallace, M. J., Yagi, N., Uesugi, K., Morgan, M. J., Hall, C., Siu, K. K. W., Williams, I. M., Siew, M., Irvine, S. C., Pavlov, K. & Lewis, R. A. (2007). *FASEB J.* **21**, 3329–3337. 10.1096/fj.07-8208com 17536040

[bb14] Kitchen, M. J., Lewis, R. A., Yagi, N., Uesugi, K., Paganin, D., Hooper, S. B., Adams, G., Jureczek, S., Singh, J., Christensen, C. R., Hufton, A. P., Hall, C. J., Cheung, K. C. & Pavlov, K. M. (2005). *Br. J. Radiol.* **78**, 1018–1027. 10.1259/bjr/13024611 16249603

[bb15] Kitchen, M. J., Paganin, D., Lewis, R. A., Yagi, N., Uesugi, K. & Mudie, S. T. (2004). *Phys. Med. Biol.* **49**, 4335–4348. 10.1088/0031-9155/49/18/010 15509069

[bb17] Lewis, R. (1997). *Phys. Med. Biol.* **42**, 1213–1243. 10.1088/0031-9155/42/7/001 9253036

[bb18] Lewis, R. A., Yagi, N., Kitchen, M. J., Morgan, M. J., Paganin, D., Siu, K. K. W., Pavlov, K., Williams, I., Uesugi, K., Wallace, M. J., Hall, C. J., Whitley, J. & Hooper, S. B. (2005). *Phys. Med. Biol.* **50**, 5031–5040. 10.1088/0031-9155/50/21/006 16237239

[bb19] Markus, M. A., Dullin, C., Mitkovski, M., Prieschl-Grassauer, E., Epstein, M. M. & Alves, F. (2014). *PLoS One*, **9**, e90017. 10.1371/journal.pone.0090017 PMC3934967 24587190

[bb20] Mohammadi, S., Larsson, E., Alves, F., Dal Monego, S., Biffi, S., Garrovo, C., Lorenzon, A., Tromba, G. & Dullin, C. (2014). *J. Synchrotron Rad.* **21**, 784–789. 10.1107/S1600577514009333 PMC4073959 24971975

[bb21] Nabe, T., Zindl, C. L., Jung, Y. W., Stephens, R., Sakamoto, A., Kohno, S., Atkinson, T. P. & Chaplin, D. D. (2005). *Eur. J. Pharmacol.* **521**, 144–155. 10.1016/j.ejphar.2005.08.015 16182277

[bb22] Paganin, D., Mayo, S., Gureyev, T. E., Miller, P. R. & Wilkins, S. W. (2002). *J. Microsc.* **206**, 33–40. 10.1046/j.1365-2818.2002.01010.x 12000561

[bb23] Parsons, D. W., Morgan, K., Donnelley, M., Fouras, A., Crosbie, J., Williams, I., Boucher, R. C., Uesugi, K., Yagi, N. & Siu, K. K. W. (2008). *J. Anat.* **213**, 217–227. 10.1111/j.1469-7580.2008.00950.x PMC2526115 19172736

[bb27] Weitoft, M., Andersson, C., Andersson-Sjöland, A., Tufvesson, E., Bjermer, L., Erjefält, J. & Westergren-Thorsson, G. (2014). *Respir. Res.* **15**, 67. 10.1186/1465-9921-15-67 PMC4089934 24950767

[bb25] Yagi, N., Suzuki, Y., Umetani, K., Kohmura, Y. & Yamasaki, K. (1999). *Med. Phys.* **26**, 2190. 10.1118/1.598735 10535637

[bb26] Yong, H. S., Kang, E.-Y., Kim, Y. K., Woo, O. H., Shin, B. K., Oh, C. H., Je, J. H., Han, H. & Seo, J. S. (2009). *Yonsei Med. J.* **50**, 422–426. 10.3349/ymj.2009.50.3.422 PMC2703767 19568606

